# Rapid, Low-Cost Dielectrophoretic Diagnosis of Bladder Cancer in a Clinical Setting

**DOI:** 10.1109/JTEHM.2020.3004743

**Published:** 2020-06-24

**Authors:** Rashedul Hoque, Hugh Mostafid, Michael Pycraft Hughes

**Affiliations:** 1 Centre for Biomedical Engineering, School of Mechanical Engineering SciencesUniversity of Surrey 3660 GuildfordGU2 7XHU.K.; 2 Royal Surrey County HospitalGuildfordGU2 7XXU.K.

**Keywords:** Dielectrophoresis, DEP, 3DEP, diagnosis

## Abstract

Bladder cancer is the 9th most common cancer worldwide. Diagnosing bladder cancer typically involves highly invasive cystoscopy, with followup monitored using uteroscopy. Molecular methods have been developed as an adjunct to this, but tend to be expensive or require expert operator input. Here we present a study of the use of dielectrophoresis (DEP) of voided cells from eight cancer-presenting patients and eight healthy controls as an alternative low-cost and operator-independent method of bladder cancer detection. This study suggests that there are statistically significant differences ($p=0.034$) between characteristics of the DEP spectrum of clinical samples, and that using this marker we were able to obtain sensitivity of 75% and specificity of 88%, in line with many molecular methods; exclusion of samples where a DEP spectrum is not present (due to low cell counts) increased sensitivity to 100%, showing this can be improved by increasing the cell collection rate. As samples were analyzed a day after collection, we suggest that the method may be amenable to a centralized mail-in analysis service.

## Graphical abstract

Diagnosing bladder cancer typically involves highly invasive cystoscopy, with followup monitored using uteroscopy. We present a study of the use of dielectrophoresis (DEP) of voided cells from 8 cancer patients and 8 controls as an alternative low-cost and operator-independent method of detection. Results demonstrate that useful DEP spectra can be obtained from the cellular components of a voided urine sample, and that these spectra can detect cancer. The test is of similar efficacy to many more expensive and complex molecular diagnostic methods.

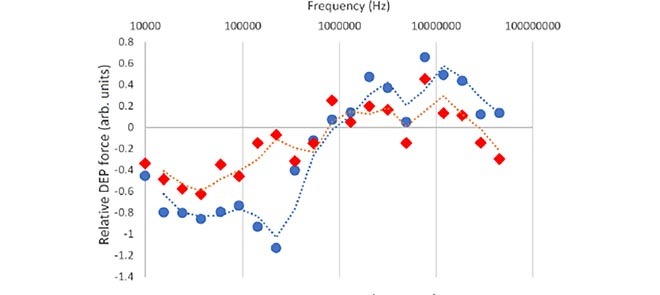

## I. Introduction

Bladder cancer (BC) is a complex disease characterized by malignant tumors arising from the tissues of the urinary bladder. BC is the seventh most common cancer in the UK, the ninth worldwide, and over 70% of BC cases being in males. The most common risk factors associated with BC are age, with the majority of cases occur in people over 60; and smoking. As the population continues to live longer, the former is likely to cause rates of incidence to increase [1], [2].

The current gold standard for diagnosing BC is cystoscopy under local anesthesia. This involves inserting a cystoscope through the urethra to observe the bladder, and taking biopsy samples. This highly invasive procedure is associated with many complications, according to the self-administered questionnaires from patients who had undergone flexible cystoscopy. Burke *et al.* [3] reported that out of the 384 returned questionnaires, 50% of patients reported pain on voiding, 37% reported urinary frequency and 19% had gross hematuria. Furthermore, Jocham *et al.* [4] showed that the sensitivity and specificity of standard white light cystoscopy is highly inconsistent, with the results of 11 studies reporting values ranging from 62-84% (mean: 73%) and 43-98% (mean: 71%) respectively. The study showed that the effectiveness of cystoscopy for detecting BC is operator dependent, with the detection of smaller tumors, satellite lesions and carcinoma in situ (CIS) being suboptimal using white light cystoscopy. Advancements in modalities such as fluorescent cystoscopy, which involves instilling a photosensitizing agent in to the bladder, have shown to improve tumor visualization, but are expensive to perform. This, coupled with the significant risk of recurrence associated with BC, makes BC one of the most expensive cancers to manage [2], [5].

At present, both urine cytology and molecular urinalysis are the only approved, non-invasive adjuncts to cystoscopy. According to a comprehensive literature review to determine the diagnostic performance of urine cytology, the modality demonstrated high specificity (>90%) but variable sensitivity, especially for low-grade tumors where sensitivity was poor (4-31%) [6]; the diagnostic accuracy of cytology is ultimately reliant on the expertise of the pathologist. There are many commercially available assays for molecular urinalysis to support cystoscopy in the screening and diagnosis of BC. For example, a study of the diagnostic performance of the marker tests NMP22 by BladderChek and ImmunoCyt on urine samples from a 109 BC patient study [7] reported that while cytology yielded the highest specificity (86%) and good sensitivity (84%) for high-grade tumors, NMP22 yielded similar specificity (85%) and the highest sensitivity (92%). ImmunoCyt was reported to have a sensitivity and specificity of 83% and 79% respectively. Sensitivity values for low-grade tumors were below 50% for NMP22 and ImmunoCyt [7]. Another molecular approach, UroVysion (a fluorescence in-situ hybridization assay) also demonstrated good sensitivity and specificity for higher grade and stage tumors, with low-grade and stage tumors reporting values as low as 30% [8]. Moreover, the relatively high cost of the assay itself and the laboratory equipment, the time-consuming nature of the procedure and the need for accurate interpretation by an experienced scientist, pose significant barriers for the use of UroVysion in routine practice [2].

Consequently, a need exists for a low-cost, sensitive and operator-independent method to discriminate between cancerous and normal cells in voided urine. It is known [9] that cancer cells express different electrophysiological parameters to equivalent healthy tissue; for example, Chuang *et al.* [10] used immortalized bladder cancer cell lines to show that lower-grade BC cells possessed different electrical properties to higher-grade BC cell lines. One method of rapid assessment of the electrical properties of cells is through the use of AC electrokinetic techniques such as dielectrophoresis (DEP) [11] and electrorotation [12]. These use the response of suspended cells to non-uniform or rotating AC electric fields across a wide frequency range, in order to determine passive electrical properties such as whole-cell membrane capacitance, conductance, and cytoplasm conductivity. These properties are related to medium conductivity and electric field frequency through a parameter called the Clausius-Mossotti factor, a frequency spectrum which describes how cells respond to a polarizing field [11]. DEP has been explored for the detection of multiple tumor types; for example, Gascoyne [13] showed that DEP could be used to isolate circulating tumor cells, a technique refined by An *et al.* [14]. Salmanzadeh *et al.* [15] demonstrated electrophysiological changes in many differentiating cells, whilst Alshareef *et al.* [16] demonstrated the separation of MCF-7 human breast cancer cells from HCT-116 colorectal cancer cells by DEP. Recent studies have shown the efficacy of DEP-Well electrode structures for rapid analysis of large samples [17], particularly using the 3DEP commercial cytometry platform [18]. This was used in the first demonstration of DEP as a clinical tool in 2015, when Graham *et al*. used DEP to discriminate between normal oral epithelium and oral and oropharyngeal cancer cells taken in brush biopsies from 57 participants. Results showed DEP offered a specificity of 81.0% and a sensitivity of 81.6% [19], suggesting that DEP could lead to swifter identification and diagnosis of oral cancer in primary care. The approach taken in the study was unusual in DEP research, due to the highly heterogeneous nature of the samples; rather than using DEP to determine the properties of the cells explicitly, the study used characteristics of the frequency dependent DEP behavior – in particular a transition frequency – to assess whether a sample contained cancerous or normal cells. This allowed the extraction of meaningful diagnostic data when faced with highly noisy spectra taken from clinical samples, without requiring additional tests such as cell radius measurements to be taken.

In this paper we present the analysis of a pilot study of 16 (8 diagnosed with bladder cancer, 8 healthy controls) participants to see whether a similar approach may be applicable to the detection of BC.

## II. Materials and Methods

### A. Participant Recruitment

Voided urine samples were taken from BC patients, diagnosed via cystoscopy, attending the Urology Outpatients at the Royal Surrey County Hospital. A total of 8 patients, 6 males and 2 females were recruited, with an average age of 73 ± 10 and 73 ± 12 respectively (mean ± standard deviation). Additionally, 8 healthy participants, 7 males and 1 female, were recruited as the control group, aged 52 ± 17 and 47 respectively. All individuals partaking in the study received a Participant Information Sheet; recruited individuals provided verbal consent and completed an Informed Consent Form. Good Clinical Practice guidelines were followed throughout the project, with clinicians completing a simple patient information slip for samples collected, anonymizing each patient by designating a unique identification number.

Samples were collected, processed, analyzed and stored in accordance to the Human Tissue Act 2005, as well as the University of Surrey ethical guidelines associated with the collection and processing of human samples [20]. This study was approved by the National Research Ethics Service (NRES) Committee London – Brent (REC reference: 13/LO/0739).

### B. Sample Processing and DEP Analysis

Following collection, urine samples were stored at 4° for 24 hours prior to analysis. DEP experimental medium was prepared by dissolving 42.5 g sucrose and 1.5 g dextrose in 500 mL of deionised water. $1250~\mu \text{L}$ of 100 mM MgCl_2_ and $500~\mu \text{L}$ of 100 mM CaCl_2_ was added to the solution. Finally, phosphate buffered saline was added to the medium whilst conductivity was measured with a Jenway 470 conductivity meter, to create a low-conductivity iso-osmotic DEP experimental medium of conductivity 43 mS.m^−1^. The DEP experimental medium was then sterilized under the laminar flow hood via filter and vacuum pump, prior to being introduced to samples. 12 mL of each sample was transferred to individual centrifuge tubes and spun down at 1400 rpm for 5 minutes. The supernatant was aspirated, disposed of in Virkon^®^ disinfectant solution, and the remaining cell pellet was suspended in the DEP experimental medium, centrifuged at 1400 rpm for 5 minutes, and suspended again in 1 mL of fresh DEP experimental medium [17], [21]. Samples were analyzed using a 3DEP cytometer (Deparator, UK). Samples were analyzed for 30 seconds between 10 kHz and 45 MHz at 10V peak-to-peak. At least three technical repeats were performed per sample; four or five were used if sufficient cells were present. Following data collection, technical repeats were averaged and analyzed in Excel (Microsoft, Redmond WA). All samples were used in the analysis.

## III. Results

**FIGURE 1.**
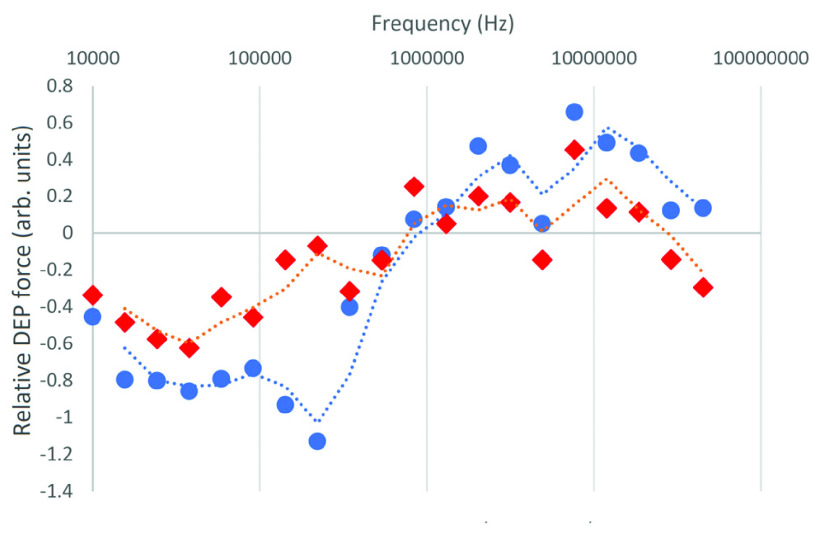
The mean DEP spectra of cells from urine samples collected from eight cancer patients (blue circles) and eight healthy controls (red diamonds). Dotted lines indicate trendlines (rolling-average with a window of 2).

When analyzed individually, many of the DEP spectra obtained from the 16 samples showed substantial amounts of noise. In order to establish underlying trends, the DEP spectra produced from all cancerous and control samples were averaged separately and compared in order to identify underlying, common characteristics in the spectra. The resultant average spectra can be seen in Fig. 1.

**FIGURE 2.**
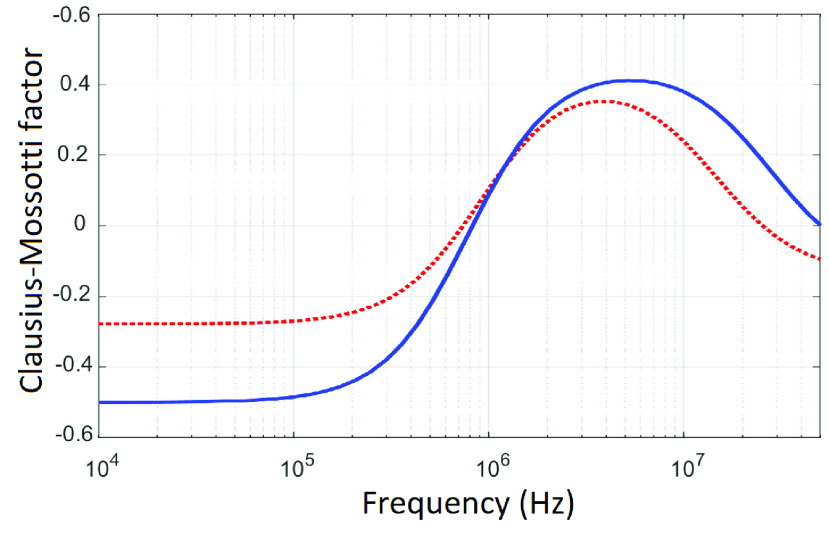
DEP spectra for a cell of arbitrary size, in two states, following similar patterns to those observed in experiments. The red broken line (similar to control cells) exhibits a higher value of membrane conductance (affecting low-frequency behavior) but lower cytoplasm conductivity (affecting high-frequency behavior) than cancer cells (blue solid line).

Whilst the level of noise remains appreciable even in the averaged spectra, it is also possible to see that the spectrum of cancerous cells exhibits a more negative value than healthy control cells at lower frequencies (below ca. 80kHz), and a more positive value than healthy samples above 100kHz. In keeping with the approach taken by Graham *et al.* [19], we did not measure cell size in order to eliminate an additional step from a putative clinical assay, and instead focused on identifying key differences in the spectrum as obtained. We can consider what differences might contribute to these differences by considering DEP spectra (and the Clausius-Mossotti factor that is shown) for cells of arbitrary size. For example, Fig. 2 shows two Clausius-Mossotti factors similar to the observed spectra; differences in these spectra suggest that the cancerous samples exhibit a much lower membrane conductance than the healthy control cells, and a higher cytoplasm conductivity, raising the points at the highest frequencies from negative to positive. Whilst this information is not used in our diagnostic test, it sheds light on the potential biophysical basis underpinning the differences in behavior.

Following the approach outlined by Graham *et al.* of identifying absolute differences in the observed spectrum, we identified that the greatest difference between the observed mean spectra were the larger absolute values observed in cancer cells at both the lower and upper ends of the DEP spectrum. Given the high level of noise observed on individual spectra, we used the means of groups of data points exhibiting the greatest degree of difference between the average cancer and control spectra. Given that cancer exhibited the lowest values below the crossover frequency (where the Clausius-Mossotti factor passes the axis to become positive) and the highest values above, we used the median values across the technical repeats to minimize the effect of noise, the averaged the spectrum values across the lowest 9 points to determine a mean “low” value, repeated this for the highest nine to obtain a mean “high” value, and classified spectra according to the difference between them (the Mean Difference Value or MDV). Point 15 was removed from the analysis due to the presence of a dead connection (visible by near-zero values in Fig. 1).

**FIGURE 3.**
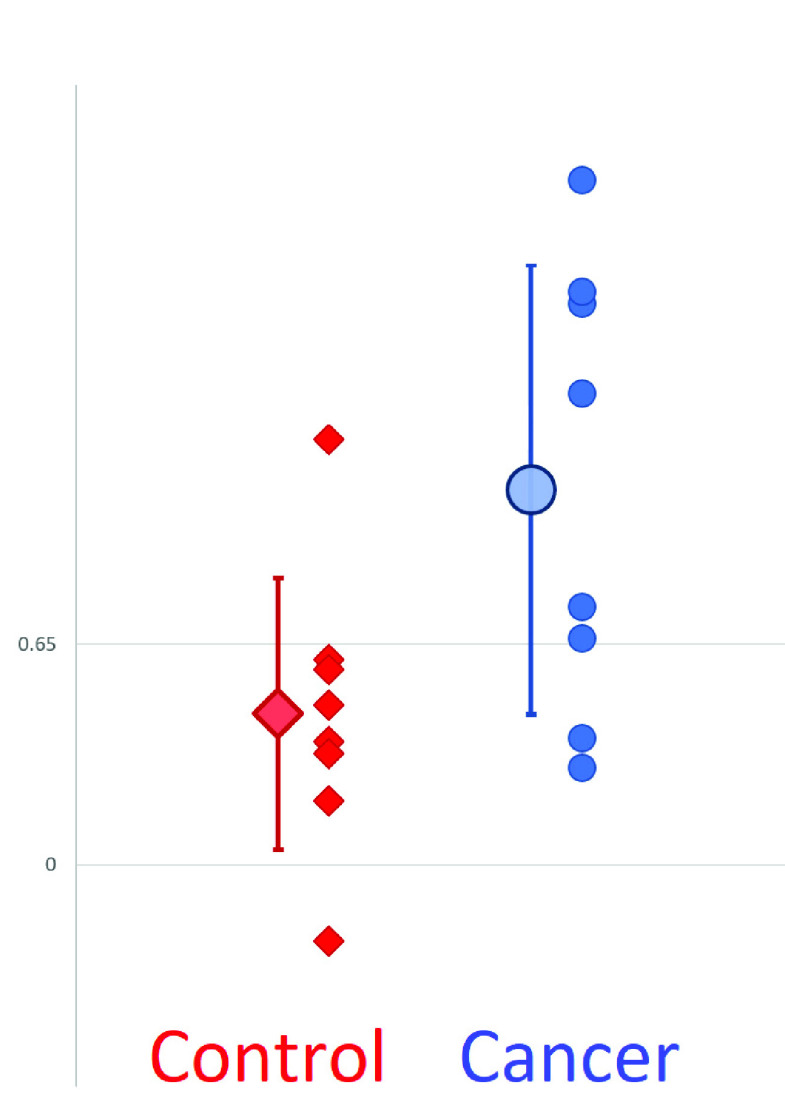
Mean Difference Values for cancerous (blue circles) and healthy control (red diamonds) cells, together with the mean and standard deviation (large markers with error bars). Optimum sensitivity and specificity were obtained when a value of 0.65 was used as a discriminator (illustrated), yielding sensitivity and specificity values of 75% and 87.5% respectively.

The distribution of the resulting difference in values can be seen in Fig. 3. We found an optimum value of sensitivity (0.75) and specificity (0.88) was obtained when cells were classified as cancerous when MDV was < 0.6-0.65, and as healthy when >0.6-0.65. A two-tailed student $t$-test suggests a significance value of $p=0.034$, indicating a statistically significant difference between the two populations.

**FIGURE 4.**
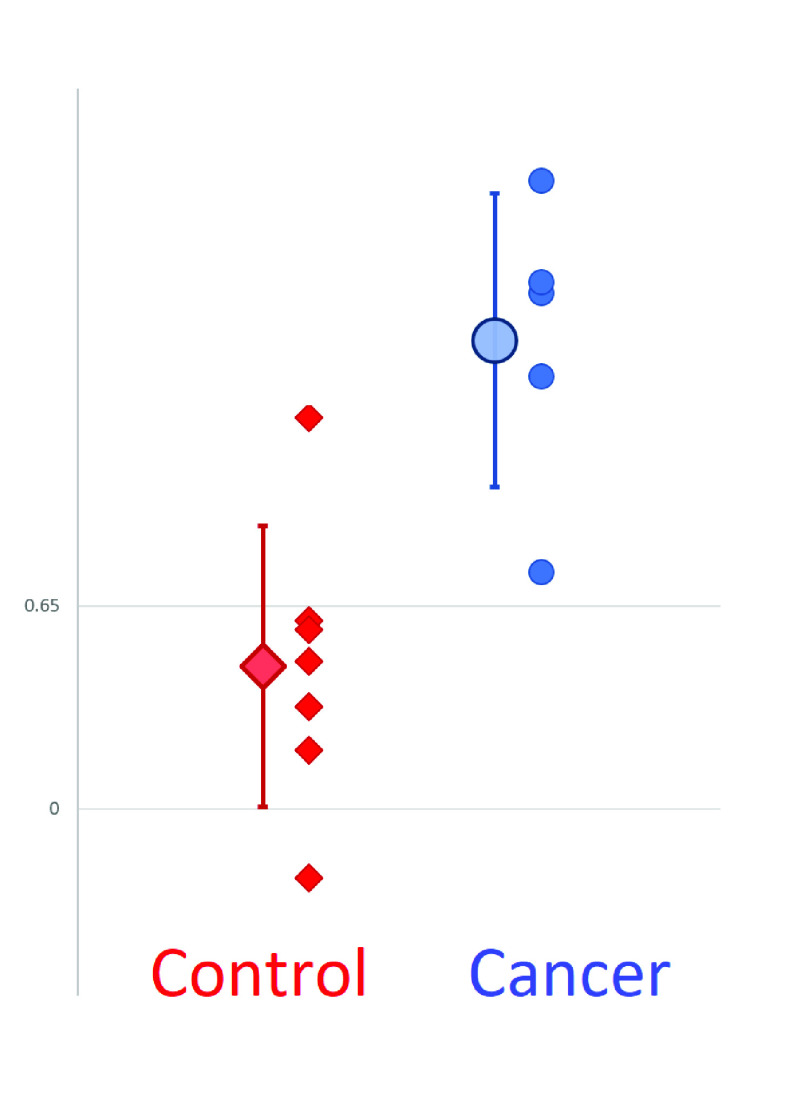
Mean Difference Values for cancerous (blue circles) and healthy control (red diamonds) cells, together with the mean and standard deviation (large markers with error bars), when 3 samples with low standard deviation (>0.4) were excluded from the study. Sensitivity was found to increase to 100% with virtually no change to specificity.

The most significant problem was a lack of cells in the sample, resulting in noisy spectra but not including an underlying DEP spectrum. In order to identify and exclude such samples, we examined the spectra of all samples and found that low cell number correlated with low values of standard deviation in the spectrum, due to the absence of the wide difference between low and high plateaux. When we eliminated samples with exceptionally low standard deviation (>0.4), we found this eliminated 3 out of 16 samples, as shown in Figure 4. When re-analyzed with these samples excluded, we found that statistical significance improved to $p=0.0038$, sensitivity rose to 1 whilst specificity remained approximately the same at 0.86. This suggests that increasing the number of cells per sample could offer improvements in performance.

## IV. Discussion

This work suggests that DEP offers potential as a rapid (total time <15min including sample preparation), low-cost (total consumable cost per test was approximately $5) diagnostic for monitoring BC. Preliminary accuracy measures are comparable to many molecular or cytometric measures whilst offering significant reductions in cost, complexity, discomfort for the patient or operator error. Analysis of the samples suggests that there is scope for further improvement. The primary source of noise was low cell count in the samples. Voided urine samples were taken due to the ease of sample acquisition and its non-invasive nature, increasing the willingness of both BC patients and healthy participants to provide samples without the fear of any adverse effects. However, the overall cell concentration observed in voided urine samples is typically less than instrumented samples, such as bladder washings. There are numerous perceived advantages of bladder washings over voided urine samples, including greater cellularity, minimal genital tract contamination and a more homogenous population of urothelial cells for analysis [8]. However, acquiring instrumented urine is not only time consuming and requires the involvement of an experienced clinician, hindering its practicality in the clinical setting, but is highly invasive and associated with complications, including hematuria and infection [22]. Moreover, instrumented urine specimens have been associated with a morphological change in otherwise normal urothelial cells, known as instrumentation artefacts, possibly causing inaccurate clinical interpretations of these cell populations [8], [23].

Alternatively, the first micturition of the day, or “first morning” specimen, is often highly cellular in its composition, which is usually attributed to a large concentration of degenerated urothelial cells. As these degenerated cells tend to not be indicative of the “true” urothelial cells, exfoliated from the urothelium of the bladder, current investigatory practices do not support conducting morphologic and biomarker analysis from these samples. However, the consensus is that morning specimens are the preferred type for any cytological examinations, with the overall cellularity of voided urine peaking in the morning and reducing as the day proceeds [8], [24]. Therefore, the collection of urine samples from an evening clinic may have reduced the cell availability reducing the sensitivity of the analysis due to lower cell concentrations. A preferred alternative to investigate would be the “second morning” voided sample, which would have an overall higher cell concentration without the complication associated with degenerated urothelial cell populations. Analysis of “second morning” voided urine specimens, collected over three consecutive days, has shown to optimize the detection of urothelial malignancy, though the practicality and financial implications of this method has made it an uncommon practice [25].

Another potential factor to consider is the impact of gender on accuracy. Female voided urine samples are subject to squamous cell contamination from the female genital tract, which may exacerbate the level of noise observed in their respective DEP spectra [8]. While substantial noise was observed in the DEP spectrum from one female sample, this was not found in all female samples and many samples from males also contained high levels of noise in their respective DEP spectra. However, the ratio of male to female samples analyzed, 6:2 (BC) and 7:1 (healthy), as well as the overall sample size of this investigation, were far too low to conduct meaningful statistical analyses on the spectral variation between genders. Of the incorrect results obtained, the single false positive was from a male donor whilst the two false negatives comprised one sample from each gender, suggesting gender is not a factor.

It is also useful to note that the analyses presented here were measured approximately 20h after collection. This suggests that the analysis may be amenable to mail-in or other centralized collection services, where a single analysis device may be able to collect and analyze samples across a large area, improving the efficiency of the method as a diagnostic tool.

## V. Conclusion

This work demonstrates that useful DEP spectra can be obtained from the cellular components of a voided urine sample, and that these spectra can discriminate between urine specimens from BC patients and healthy participants. A statistically significant difference was found in the mean difference between average high and average low values, characterized by the Mean Difference Value or MDV. Analysis of the results suggests the test is of similar efficacy to many more expensive and complex molecular diagnostic methods.

A limitation of this study was the small sample size, and elevated levels of noise in the DEP spectra due to low sample numbers collected in voided urine. Methods to increase assay sensitivity, such as collecting “second morning” voided specimens. Subsequent study and refined sample collection and analysis protocols will be used to improve this in subsequent trials.

This study demonstrates for the first time that DEP analysis can be performed on cells harvested from urine specimens, and that DEP-based techniques have potential for low-cost, rapid and operator-independent cancer diagnosis across a range of tumors beyond oral cancer. It is hoped that subsequent analyses will identify even broader applicability of the technique.
